# Evaluation of perturbed iron-homeostasis in a prospective cohort of patients with COVID-19

**DOI:** 10.12688/wellcomeopenres.17904.1

**Published:** 2022-06-21

**Authors:** Joe N. Frost, Fergus Hamilton, David Arnold, Karen T. Elvers, Akshay Shah, Andrew E. Armitage, Alice Milne, Jorgen McKernon, Marie Attwood, Yi-Ling Chen, Luzheng Xue, Jonathan Youngs, Nicholas M. Provine, Tihana Bicanic, Paul Klenerman, Hal Drakesmith, Peter Ghazal

**Affiliations:** 1 MRC Human Immunology Unit, MRC Weatherall Institute of Molecular Medicine, University of Oxford, Oxford, OX1 2JD, UK; 2 MRC Integrative Epidemiology Unit, University of Bristol, Bristol, BS10 5NB, UK; 3 North Bristol NHS Trust, Bristol, BS10 5NB, UK; 4 Medicines Discovery Institute, Cardiff University, Cardiff, UK; 5 Nuffield Department of Clinical Neurosciences, University of Oxford, Oxford, UK; 6 Respiratory Medicine Unit and Oxford NIHR Biomedical Research Centre, Nuffield Department of Medicine, University of Oxford, Oxford, UK; 7 Institute for Infection and Immunity, St George's, University of London, London, UK; 8 Clinical Academic Group in Infection and Immunity, St George's Hospital, London, London, UK; 9 Peter Medawar Building for Pathogen Research, University of Oxford, Oxford, UK; 10 Translational Gastroenterology Unit, Nuffield Department of Medicine, University of Oxford, Oxford, UK; 11 Project Sepsis, Systems Immunity Research Institute, Division of Infection and Immunity, Cardiff University, Cardiff, UK

**Keywords:** iron, COVID-19, homeostasis, ferritin, haemoglobin

## Abstract

**Background: **Marked reductions in serum iron concentrations are commonly induced during the acute phase of infection. This phenomenon, termed hypoferremia of inflammation, leads to inflammatory anemia, but could also have broader pathophysiological implications. In patients with coronavirus disease 2019 (COVID-19), hypoferremia is associated with disease severity and poorer outcomes, although there are few reported cohorts.

**Methods: **In this study, we leverage a well characterised prospective cohort of hospitalised COVID-19 patients and perform a set of analyses focussing on iron and related biomarkers and both acute severity of COVID-19 and longer-term symptomatology.

**Results: **We observed no associations between acute serum iron and long-term outcomes (including fatigue, breathlessness or quality of life); however, lower haemoglobin was associated with poorer quality of life. We also quantified iron homeostasis associated parameters, demonstrating that among 50 circulating mediators of inflammation IL-6 concentrations were strongly associated with serum iron, consistent with its central role in inflammatory control of iron homeostasis. Surprisingly, we observed no association between serum hepcidin and serum iron concentrations. We also observed elevated erythroferrone concentrations in COVID-19 patients with anaemia of inflammation.

**Conclusions: **These results enhance our understanding of the regulation and pathophysiological consequences of disturbed iron homeostasis during SARS-CoV-2 infection.

## Introduction

Systemic hypoferremia commonly occurs during the acute phase of infection ( Drakesmith & Prentice, 2012) and is well-established to contribute to the anaemia of inflammation. However, iron is not only important for erythropoiesis and plays essential roles in cellular biochemistry ( Andreini *et al*., 2018). In animal models reduced iron availability can antagonise the development of a protective antiviral immune response ( Frost *et al*., 2021) and influence tissue repair ( Recalcati *et al*., 2019). 

We have previously demonstrated that serum iron concentration within 24 hours of a critical care admission is inversely associated with disease severity in coronavirus disease 2019 (COVID-19), a result which has also subsequently been confirmed in other cohorts including a wider range of disease severities ( Hippchen *et al*., 2020; Lv *et al*., 2021; Moreira *et al*., 2021; Shah *et al*., 2020; Zhao *et al*., 2020). Elevated levels of the iron regulatory hormone hepcidin ( Nai *et al*., 2021) and anaemia ( Bellmann-Weiler *et al*., 2020) have also been associated with worse outcomes. This contrasts with findings in sepsis where low serum iron concentrations may be associated with better outcomes ( Brandtner *et al*., 2020; Lan *et al*., 2018; Tacke *et al*., 2016).

It remains unclear to what extent perturbed iron status, at an early time point, may predict later outcomes such as survival in COVID-19. Furthermore only a few studies have considered if iron status is perturbed long term in COVID-19 patients ( Sonnweber *et al*., 2020), and whether disturbed iron status plays a role in the persistent symptoms a proportion of patients often continue to suffer with, such as fatigue and difficulty concentrating ( Carfì *et al*., 2020). Furthermore, multiple physiological inputs and regulatory factors (inflammation, iron status, hypoxia and erythropoietic drive) can potentially influence systemic iron status during inflammation both, dependent and independent, of serum hepcidin – the master regulator of iron status ( Weiss *et al*., 2019). Given the important role of iron in immunity and erythropoiesis, an understanding of the factors driving disturbed iron homeostasis in COVID-19 is necessary.

We undertook deep phenotyping of iron homeostasis by integrating data between two prospective cohorts of COVID19 patients to investigate: (i) associations between patient severity and outcome with iron parameters; (ii) immunological factors associating with altered iron homeostasis during the acute stages of infection and; (iii) the extent to which iron status is perturbed in convalescence.

## Methods

### Cohorts

This was a retrospective analysis based on data collected prospectively. We report our findings in accordance with STROBE guidance.

In this study, two cohorts of patients were recruited. Patients in the DISCOVER (DIagnostic and Severity markers of COVID-19 to Enable Rapid triage) cohort were prospectively recruited at two NHS sites, North Bristol NHS Trust and Gloucester Royal Infirmary. Detailed description of the inclusion, exclusion, criteria and a summary of patient characteristics are available with the original publication ( Arnold *et al*., 2021a). Ethical approval was given by the South Yorkshire REC (Ref: 20/YH/0121). Briefly, patients who presented to either site were prospectively recruited with either polymerase chain reaction (PCR) confirmed or clinically suspected COVID-19. Clinical details were extracted from the medical notes and blood sampling was taken as soon as possible after recruitment, usually on the day of recruitment. Acute outcomes were recorded in line with the RECOVERY trial ( Abani *et al*., 2021), and included intensive care utilisation, oxygen requirements, and mortality. Severity was defined as severe (requirement for ITU admission, non-invasive ventilation, or death), moderate (requirement for oxygen only), or mild (no requirement for oxygen), or using a binary outcome of severe disease (NIV, ITU, or death), as per Arnold *et al*. (2021a). Patients were subsequently followed up in person at 3 and 9 months to assess functional recovery; details of these assessments are provided here ( Arnold *et al*., 2021b).

A second cohort of severe COVID-19 patients, termed the AspiFlu cohort previously published in ( Youngs *et al*., 2021), provided serum for iron and cytokine analysis. The prospective observational study AspiFlu (ISRCTN51287266) has national HRA (CPMS 43440/IRAS 271269) and REC (19/WA/0310) approval.

### Serum analysis

Serum iron in both cohorts was quantified using the Abbott Architect c16000 automated analyser (Abbott Laboratories) and the Abbott MULTIGENT Iron Kit (6K95-30) at Oxford John Radcliffe Hospital, UK.

Erythroferrone (ERFE) and serum hepcidin were measured in patient serum from the DISCOVER cohort using Intrinsic Erythroferrone IE ELISA Kit (ERF-001) and Intrinsic Hepcidin IDx ELISA Kit (ICE-007), respectively, in accordance with the manufacturer’s instructions.

Details on sample preparation and serum cytokine measurements for the AspiFlu cohort are available in the original publication ( Youngs *et al*., 2021).

### Statistical analysis

For visualisation in the DISCOVER dataset, one way ANOVA or Welches T-test conducted in GraphPad Prism version 9 (RRID:SCR_002798) was used on log-transformed parameters. R (R Foundation, Vienna) could also be used to replicate this analysis. For all downstream analysis, log-transformation was performed when data was visually log-normal.

For association of iron and haemoglobin status on admission and the binary outcome of severe disease and/or death, we used logistic regression in both unadjusted analyses, and adjusted for age and sex. To estimate the associations across iron markers and other inflammatory biomarkers, Pearson’s correlation was calculated the cor function in R v 4.0.4 Project for Statistical Computing (RRID:SCR_001905).

Linear regression was used to estimate the effect on continuous outcomes (e.g. quality of life metrics), adjusting for age and sex. Analysis was performed using the lm function in R with the “tidyverse” package used for data manipulation and plotting, while correlation plots and matrixes were generated using ggcorrplot. We performed a complete-case analyses for all analyses.

For the ASPIFLU cohort, Spearmen’s R (non-parametric correlation) and adjusted p value (Holm-Sidak alpha = 0.05) were calculated in Graph Pad Prism for analysis of associations between serum cytokines (each with different distributions) and serum iron.

## Results

In total, 321 participants were recruited to DISCOVER, of which 246 participants had serum stored and had at least one analysis of an iron biomarker performed. Table 1 describes this cohort, stratified by disease severity. The cohort was middle aged (median age 59, IQR 47-74), and had a male predominance (59% male). The majority had proven COVID-19, with 15% having suspected COVID-19 with negative PCR testing. Common to other UK cohorts, comorbidities were common and higher in those with severe disease, with 25% of patients having chronic lung disease ( Hamilton, 2022).

**Table 1.** Characteristics of the DISCOVER cohort.

Characteristic	Mild, N = 58	Moderate, N = 146	Severe, N = 42	p-value
**Age**	**54 (36, 67)**	**59 (47, 74)**	**62 (55, 75)**	**0.002**
**Unknown**	**0**	**1**	**0**	
**Sex**				**0.014**
**Male**	**26 (45%)**	**97 (66%)**	**23 (55%)**	
**Female**	**32 (55%)**	**49 (34%)**	**19 (45%)**	
**Proven or suspected COVID-19**				**0.028**
**Proven**	**43 (74%)**	**129 (89%)**	**36 (86%)**	
**Suspected**	**15 (26%)**	**16 (11%)**	**6 (14%)**	
**Unknown**	**0**	**1**	**0**	
**Inpatient or outpatient on recruitment**				**<0.001**
**Inpatient**	**48 (83%)**	**140 (96%)**	**42 (100%)**	
**Outpatient**	**10 (17%)**	**6 (4.1%)**	**0 (0%)**	
**adm_diabetes.factor**				**>0.9**
**No**	**48 (83%)**	**124 (85%)**	**32 (80%)**	
**Type 1 diabetes**	**1 (1.7%)**	**3 (2.1%)**	**1 (2.5%)**	
**Type 2 diabetes**	**9 (16%)**	**19 (13%)**	**7 (18%)**	
**Unknown**	**0**	**0**	**2**	
**Heart disease?**	**9 (16%)**	**32 (22%)**	**10 (25%)**	**0.5**
**Unknown**	**0**	**2**	**2**	
**Chronic Lung disease?**	**8 (14%)**	**33 (23%)**	**20 (49%)**	**<0.001**
**Unknown**	**2**	**1**	**1**	
**Severe Liver disease?**	**1 (1.7%)**	**4 (2.8%)**	**0 (0%)**	**0.8**
**Unknown**	**0**	**1**	**0**	
**Severe kidney impairment (eGFR< 30 or dialysis)**	**4 (6.9%)**	**14 (9.7%)**	**2 (4.8%)**	**0.7**
**Unknown**	**0**	**2**	**0**	
**Hypertension?**	**13 (24%)**	**30 (23%)**	**14 (34%)**	**0.3**
**Unknown**	**3**	**15**	**1**	
**HIV on admission**	**1 (1.7%)**	**2 (1.4%)**	**1 (2.4%)**	**0.8**
**Unknown**	**0**	**1**	**0**	
**Non white ethnicity**	**2 (6.2%)**	**12 (15%)**	**4 (19%)**	**0.4**
**Unknown**	**26**	**64**	**21**	
**Serum iron (umol/L)**	**9.0 (4.5, 12.3)**	**7.0 (4.9, 11.2)**	**5.0 (4.0, 8.8)**	**0.03**
**Unknown**	**7**	**16**	**3**	
**Serum UIBC (umol/L)**	**38 (30, 46)**	**32 (26, 37)**	**31 (25, 40)**	**0.003**
**Unknown**	**7**	**16**	**3**	
**Serum TIBC (umol/L)**	**48 (40, 54)**	**39 (35, 45)**	**39 (32, 46)**	**<0.001**
**Unknown**	**7**	**16**	**3**	
**ERFE (ng/mL)**	**2.0 (1.2, 5.8)**	**1.9 (1.3, 3.6)**	**3.0 (0.8, 8.0)**	**0.7**
**Unknown**	**43**	**93**	**28**	
**Hepcidin (ng/ml)**	**133 (87, 221)**	**350 (206, 458)**	**242 (142, 401)**	**0.005**
**Unknown**	**39**	**85**	**27**	
**Haptoglobin (g/L)**	**2.35 (1.55, 3.40)**	**3.63 (2.95, 4.30)**	**3.80 (3.16, 4.32)**	**<0.001**
**Unknown**	**9**	**20**	**3**	
**1. Median (IQR); n (%)** ** 1 Kruskal-Wallis rank sum test; Pearson's Chi-squared test; Fisher's exact test**				

### Higher serum iron concentration associates with better outcomes and survival following severe acute respiratory syndrome coronavirus 2 (SARS-CoV-2) infection

**Figure 1.**
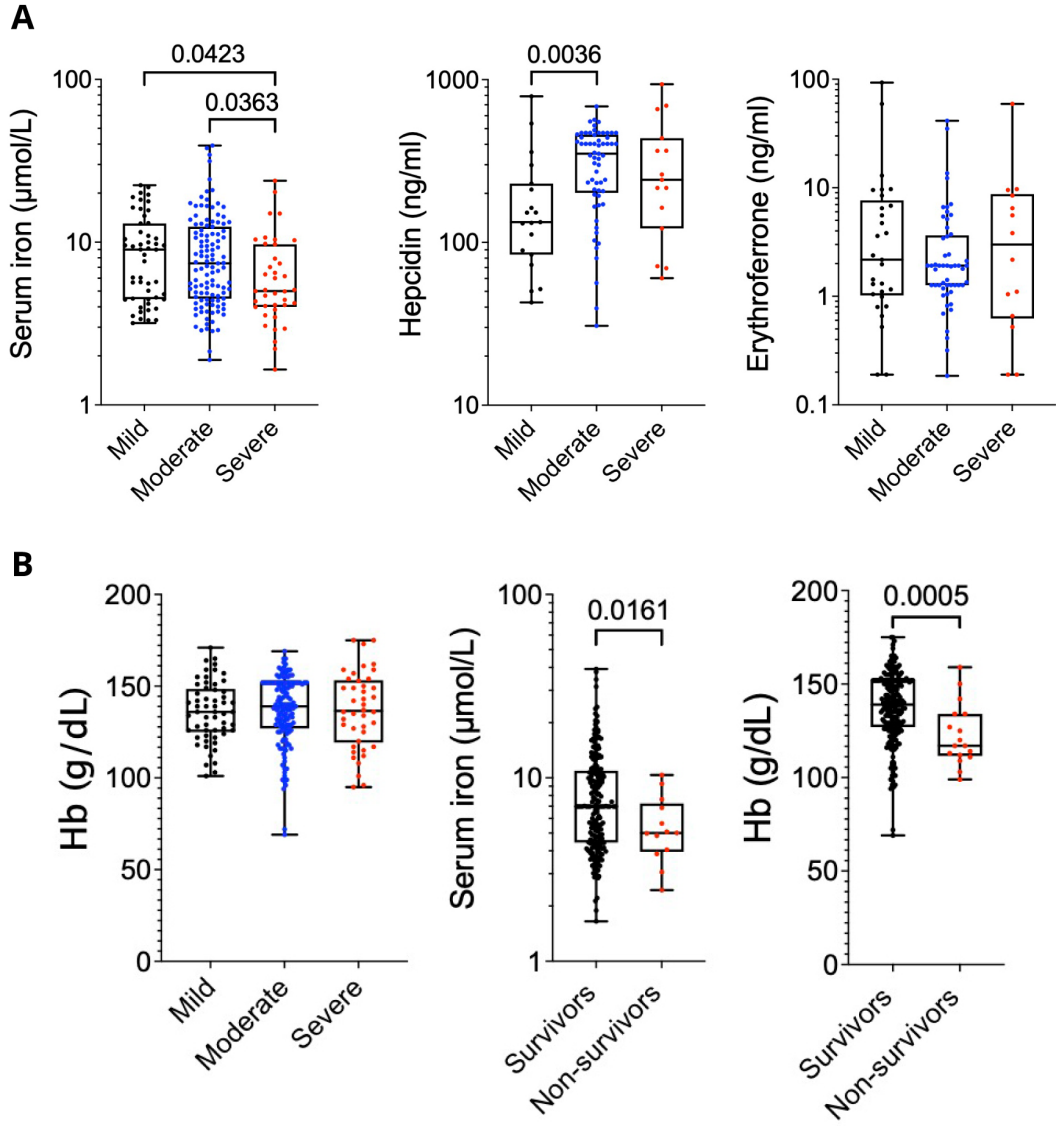
( **A**) Serum iron, hepcidin and erythroferrone in DISCOVER cohort split by severity. One way ANOVA on log transformed data. Median, upper/ lower quartiles and range. ( **B**) Serum iron split by outcome. Welches T-test. Hb split by outcome. Mann-Whitney Test. Median, upper/ lower quartiles and range.

Serum iron concentrations within 24 hours of admission in the DISCOVER cohort were profoundly decreased in all severity groups relative to the normal patient range of 10-30µmol/L ( Table 1, Figure 1) ( Ritchie *et al*., 2002). Analysis by ANOVA with Tukey’s multiple comparisons highlighted that serum iron levels were suppressed in patients with the most severe disease, with hepcidin elevated in patients with moderate or severe disease relative to mild presentation.

Using the previous binary outcome of severe disease and/or death ( Arnold *et al*., 2021a), lower serum iron was associated with worse outcomes (OR 0.42 for each increase in logged serum iron; 95% CI 0.22-0.78, p= 0.008), with a similar effect size in the analyses adjusted for age and sex (OR 0.47; 95% CI 0.24-0.85, p = 0.017), although we did not identify any association with haemoglobin (both p > 0.1).

Haemoglobin and Erythroferrone (ERFE) showed no significant differences across disease presentation states ( Figure 1A) suggesting that disturbed erythropoiesis does not associate with disease severity. Whilst the DISCOVER cohort had a low mortality, patients who did not survive had significantly lower serum iron levels and haemoglobin at admission ( Figure 1B). Our data confirms in a prospective trial setting that serum iron levels are lower in patients with the most adverse outcomes from COVID-19 infection. 

### Interactions between inflammatory biomarkers, cytokines and iron status

**Figure 2.**
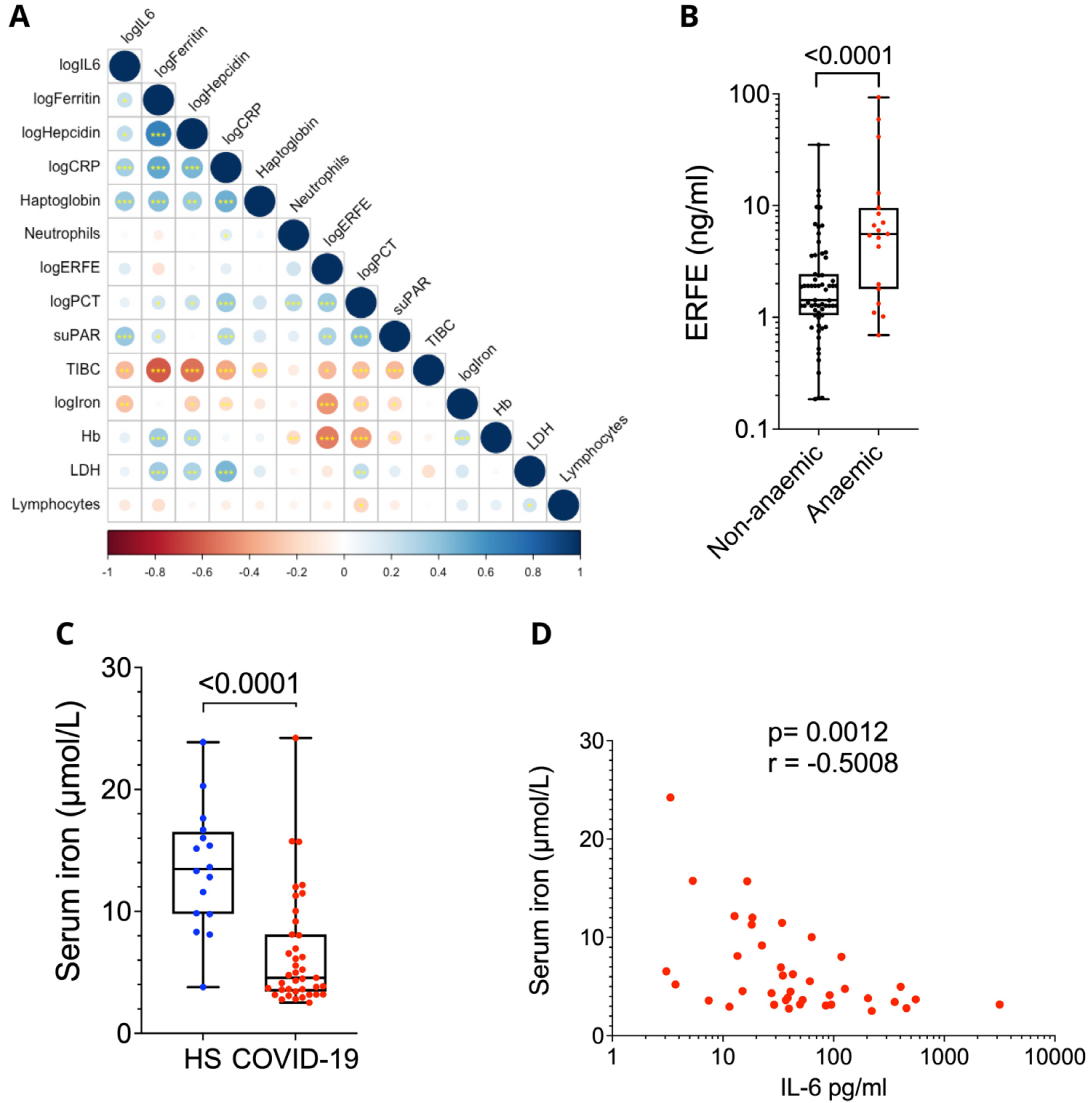
( **A**) Correlation matrix for each biomarker. The colour and size are determined by the correlation coefficient, while the number of stars determines the signifance by Pearson’s correlation. Significance stars at 0.05, 0.01, 0.001. ( **B**) Circulating ERFE split by anaemia. T-test on logged data. Median, upper/ lower quartiles and range. Anaemia defined as a Hb below 12g/dL in females and 13g/dL in males. ( **C**) Serum iron concentration in AspiFlu cohort comparing patients with healthy controls. Mann-Whitney Test. Median, upper/ lower quartiles and range. ( **D**) Correlation between IL-6 and serum iron in AspiFlu cohort. Spearman’s correlation co-efficient.

To understand the factors controlling iron homeostasis in this cohort we calculated associations for selected iron parameters (Haemoglobin, Haptoglobin, Lactate Dehydrodgenase (LDH), serum iron, total iron binding capacity, ferritin, hepcidin and ERFE) and selected inflammatory markers (CRP, IL-6, suPAR, PCT, neutrophil and lymphocyte count) across all severities. A correlation matrix is shown in Figure 2, with only nominally significant results (p <0.05) shown. A table in the *Extended data* shows all correlation coefficients and associated P values ( Hamilton, 2022).

Serum iron and IL-6 had a negative association in line with the role of IL-6 in controlling the acute phase response (Pearson’s correlation, R -0.29, p = 0.003) ( Nemeth *et al*., 2004); similar results were observed for CRP (R = -0.14, p = 0.007) consistent with both being prototypical acute phase proteins in humans. Hepcidin levels were negatively associated with serum iron (R = -0.24, p = 0.04), but were as expected positively associated with both IL-6 (R = 0.25, p = 0.03) and CRP (R = 0.46, p = 3 x 10 ^-6^), although the association was much stronger with CRP.

We also identified an association between other inflammatory markers and iron status, with both suPAR (R = -0.19, p = 0.03), and procalcitonin (R = -0.24, p = 0.009) having negative associations with iron status. As far as we are aware, this is the first reported association between procalcitonin and serum iron.

Despite its role as a negative regulator of hepcidin ( Kautz *et al*., 2014), ERFE did not associate with hepcidin in COVID-19 patients with inflammation, despite a strong association with iron (R = -0.45, p = 2 × 10 ^-4^). Unexpectedly, patients with the lowest serum iron had the highest ERFE (one may expect ERFE to drive increased serum iron through hepcidin suppression) causing us to consider the upstream control of ERFE. ERFE levels were highest in the patients with the lowest haemoglobin (R = -0.43, p = 9 × 10 ^-7^) supporting the hypothesis that anaemia leading to increased erythropoietic activity may be contributing to raised ERFE in this patient cohort (2B). Consistent with the proposal that ERFE levels reflect anaemia and therefore negatively associated with serum iron, Hb and serum iron were also positively correlated (R = 0.25, p = 2 × 10 ^-4^).

Multiple inflammatory cytokines (including IL-6, type I interferons, IL-22) have been proposed to induce hepatic hepcidin production to reduce serum iron, but the relative importance of these cytokines for particular infections *in vivo* is unknown ( Armitage *et al*., 2011; Nemeth *et al*., 2004; Ryan *et al*., 2012). In parallel to our analysis of outcomes, biomarkers and iron homeostasis in the DISCOVER cohort we measured serum iron concentration in samples from the deeply immunophenotyped AspiFlu study ( Youngs *et al*., 2021) to ask which serum inflammatory analytes during severe COVID-19 associated with serum iron suggesting possible links with its regulation. Patients in the critically ill hospitalised AspiFlu cohort showed significantly reduced serum iron compared to healthy controls ( Figure 2C). Among 50 serum analytes we found that only IL-6 presented a near significant p-value after multiple comparisons testing (p= 0.0583), with an unadjusted p-value of 0.0012 and r = -0.5008 ( Table 2, Figure 2D) highlighting IL-6 as a likely major driver of hypoferremia during human COVID-19.

**Table 2.** Serum analyte and p value (Spearmans r) for correlation with iron and adjusted p value (Holm-Sidak alpha = 0.05) for patient samples from the AspiFlu trial cohort.

Analyte	P value (Spearman’s r)	Adjusted P Value
IL-6	0.0012	0.0583
S100A9	0.0149	0.5208
CD163	0.0206	0.6318
CXCL1/GRO alpha/KC/CINC-1	0.0242	0.6838
Myeloperoxidase/MPO	0.0295	0.7478
Complement Component C5a	0.0591	0.9355
Lactoferrin	0.0857	0.9806
G-CSF	0.1295	0.9974
TREM-1	0.1312	0.9974
IL-33	0.1346	0.9974
beta-NGF	0.1361	0.9974
CCL11/Eotaxin	0.1441	0.9977
CCL18/PARC	0.1659	0.999
Coagulation Factor III/Tissue Factor	0.1691	0.999
IL-23	0.1698	0.999
CCL19/MIP-3 beta	0.173	0.999
TFPI	0.19	0.9992
FGF basic/FGF2/bFGF	0.2	0.9994
IFN-gamma	0.2091	0.9995
CXCL5/ENA-78	0.21	0.9995
Granzyme B	0.2473	0.9998
IL-5	0.2864	0.9999
CCL3/MIP-1 alpha	0.3194	1
TNF-alpha	0.3392	1
Lipocalin-2/NGAL	0.3706	1
CCL20/MIP-3 alpha	0.379	1
Thrombopoietin/Tpo	0.4383	1
IL-15	0.4442	1
IFN-alpha	0.4597	1
IL-1 beta/IL-1F2	0.4942	1
Oncostatin M/OSM	0.5238	1
CCL2/JE/MCP-1	0.5513	1
IL-2	0.5631	1
CCL17/TARC	0.5996	1
CD40 Ligand/TNFSF5	0.6407	1
IL-12 p70	0.6539	1
SCGF/CLEC11a	0.67	1
CCL4/MIP-1 beta	0.6987	1
GM-CSF	0.7543	1
TGF-alpha	0.7887	1
IL-3	0.794	1
IL-1 alpha/IL-1F1	0.798	1
M-CSF	0.8041	1
IL-17/IL-17A	0.8277	1
IL-8/CXCL8	0.8295	1
IL-13	0.8341	1
IL-10	0.8373	1
EGF	0.8506	1
CXCL10/IP-10/CRG-2	0.96	1
IFN-beta	0.9796	1

### Serum iron during acute infection does not robustly associate with long term quality of life metrics

Returning to the DISCOVER cohort, we prospectively collected quality of life data at 3,8, and 12 months using the validated SF-36 questionnaire and explored potential associations between hypoferraemia and quality of life.

**Figure 3.**
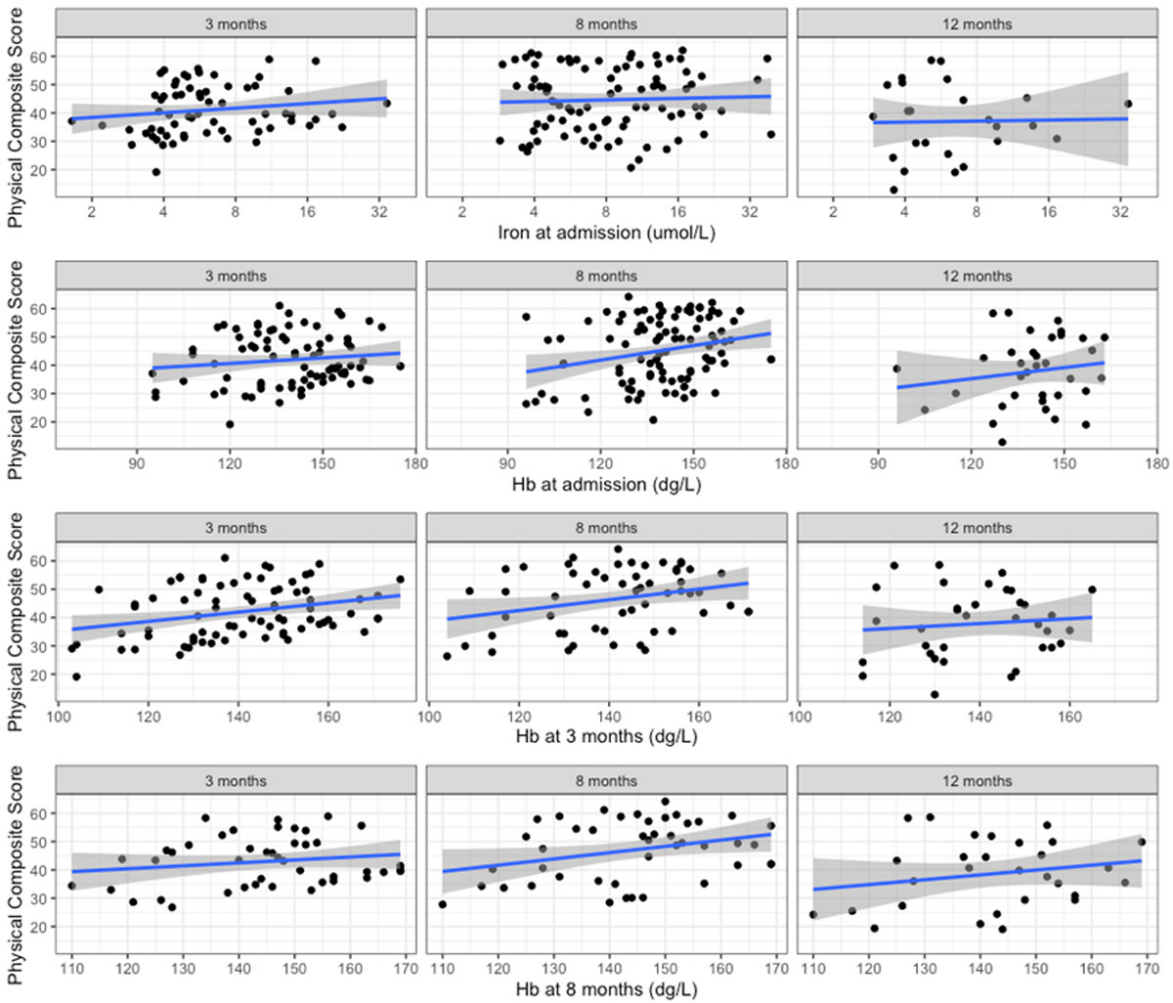
Physical Composite Score component of SF-36 vs iron biomarkers. ( **A**) Iron at admission, ( **B**) Hb at admission, ( **C**) Hb at 3 months, ( **D**) Hb at 8 months.

**Figure 4.**
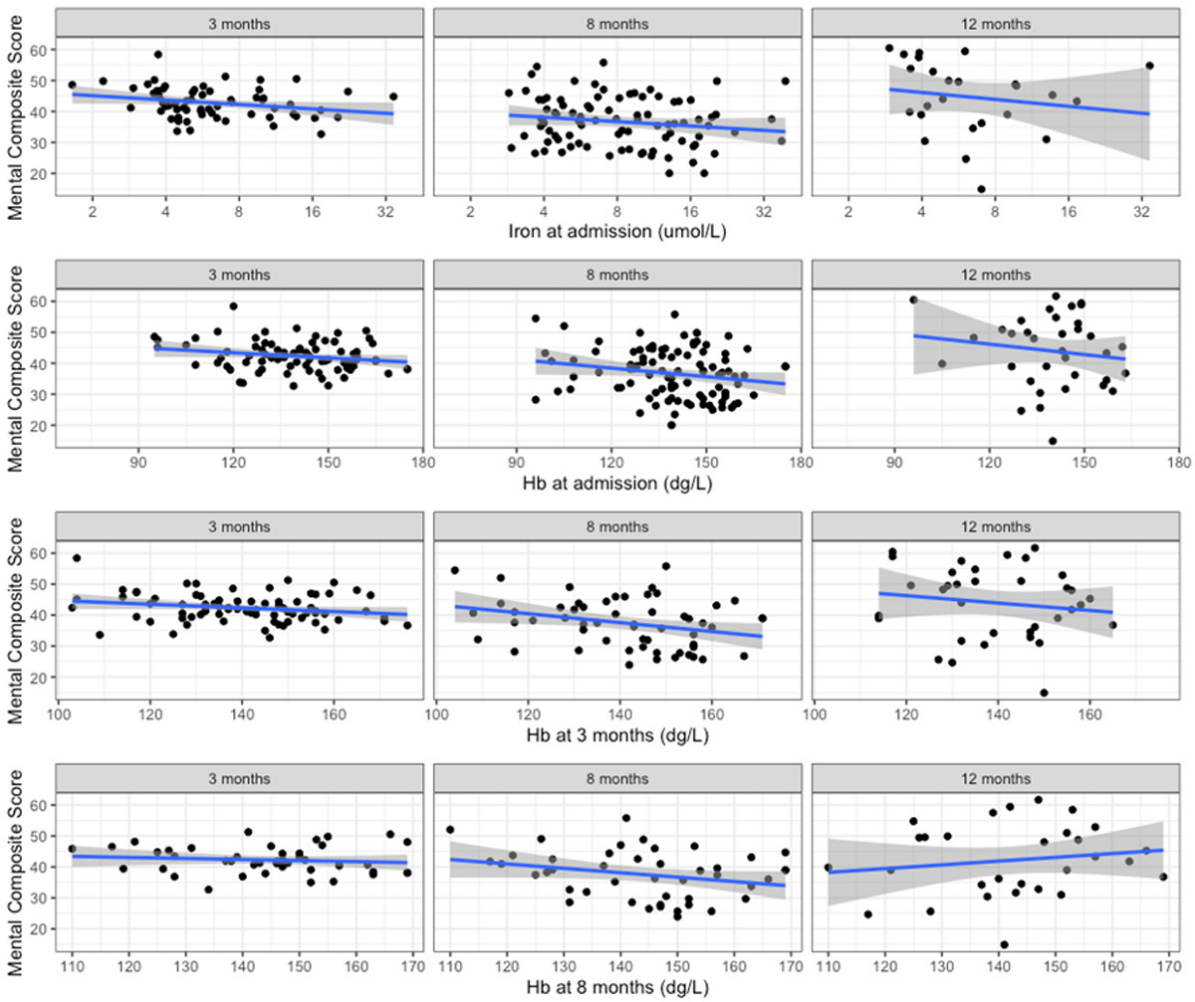
Mental Composite Score component of SF-36 vs iron biomarkers. ( **A**) Iron at admission, ( **B**) Hb at admission, ( **C**) Hb at 3 months, ( **D**) Hb at 8 months.

In logistic regression adjusted for age and sex, we found no association with serum iron at admission (logged to normalise the data) and physical composite scores ( Figure 3A, all p values > 0.4). However, for mental ( Figure 4A.) composite scores, we identified a weak association with increasing serum iron and reduced mental composite scores (beta -5.31 reduction in MSC score per log10 increase in serum iron, p = 0.04 at 3 months, - 6.39, p = 0.06) at 8 months, and -18.8, p = 0.15) at 12 months).

In univariate regression, admission haemoglobin had a positive association with physical and composite score at 8 months (beta 0.17, p = 0.01, Figure 3b), but when adjusted for age and sex, this association was lost (beta 0.08, p = 0.21). We did not identify any robust associations with the mental composite score and initial Hb, although all estimates suggested a negative correlation, in contrast to the physical composite scores ( Figure 4B).

In regression adjusted for age and sex, Hb measured at 3 months was associated with physical composite score at this time (beta 0.09, p = 0.03), suggesting that haemoglobin status is more relevant for longer term recovery than initial iron status during acute infection ( Figure 3C and D). Again, we found in univariate regression an association between Hb measured at 8 months and 8 month physical composite score (beta 0.22, p = 0.04), but this effect did not hold once adjusting for age and sex (beta 0.14, p = 0.25)

In summary, we did not identify any strong association between serum iron levels and functional status at 8 months, but did identify a weak and unexpected association with increasing iron status and reduced mental composite score.

Alongside this, we identified an expected association between haemoglobin and the physical composite score. Once adjusting for age and sex, which are known predictors of both haemoglobin and quality of life, the effect of Hb was not relevant.

## Discussion

Here, we have undertaken a deep phenotypic characterisation of iron homeostasis upon admission in a cohort of COVID-19 patients presenting with a range of severities in illness. In line with other published COVID-19 studies ( Hippchen *et al*., 2020; James *et al*., 2021; Lv *et al*., 2021; Shah *et al*., 2020; Zhao *et al*., 2020) we observe lower serum iron upon admission of patients presenting with more severe disease, despite variability between studies regarding sampling time and how disease severity is defined. This is consistent with experimental studies showing that replete serum iron levels promotes antiviral immunity ( Frost *et al*., 2021)

 Our investigation confirms in a prospective observational study that those patients who did not survive exhibited, on admission, significantly lower serum iron ( Zhao *et al*., 2020) and haemoglobin ( Bellmann-Weiler *et al*., 2020; Faghih Dinevari *et al*., 2021; Oh *et al*., 2021; Tao *et al*., 2021; Tremblay *et al*., 2021). We did not consider the aetiology of anaemia at this time point as this is difficult to characterise in a setting of severe acute inflammation. Further work is required to understand how the iron levels dynamically shift during SARS-CoV-2 infection in relation to therapy and in the context of disease progression ( Bolondi *et al*., 2020; Chakurkar *et al*., 2021; Hippchen *et al*., 2020). We were unable to identify any robust association between serum iron and persistent symptoms or quality of life after COVID-19 but did find an unexpected association with serum iron and the mental component of the SF-36 quality of life metrics.

### Limitations

This study has several limitations. Firstly, the cohorts included are small, and they were sample at a time without widespread vaccination. Secondly, serum iron is also a predictor of severity of disease, therefore the selected cohort (that by nature were alive at follow up) may be biased with respect to iron status.

Further phenotyping of patients at follow-up time points will be required to identify the factors driving anaemia and the therapeutic utility of iron supplementation in this setting. Treating anaemia of inflammation, particularly during the recovery phase of critical illness, with intravenous iron has been shown to be efficacious ( Shah *et al*., 2022) but there are no comparable data in patients recovering from COVID-19.

Multiple inflammatory mediators have been proposed to control iron status, but their relative importance in different human infections remains unclear. Our comprehensive profiling of serum cytokines highlights IL-6, above 50 other inflammatory mediators, as a likely driver of hypoferraemia in COVID-19; the strong association of IL-6 with serum iron is confirmed in our prospective cohort. IL-6 is the canonical regulator the acute phase response, well established to control hepcidin and serum iron levels ( Nemeth *et al*., 2004) and has been previously reported to associate with serum iron in COVID-19 ( Hippchen *et al*., 2020; Lv *et al*., 2021; Moreira *et al*., 2021). This finding is particularly interesting in light of the clinical evidence for efficacy of tocilizumab in COVID-19 patients ( Abani *et al*., 2021; The REMAP-CAP Investigators, 2021).

Alongside Hippchen *et al* we only observed a weak association between serum hepcidin and serum iron, at odds with the strong association observed in experimental *Salmonella typhi* and *Plasmodium falciparum* challenge ( Darton *et al*., 2015; Spottiswoode *et al*., 2017). In part this could be the age, frailty, variable iron stores and relatively high frequency of anaemia observed in COVID-19 cohorts. In addition it is possible inflammation is driving reduced serum iron in a hepcidin-independent manner ( Guida *et al*., 2015). We also highlight a novel negative association between both procalcitonin and suPAR, and serum iron. Positive associations between ferritin and serum iron suggest that even in COVID patients with elevated ferritin due to inflammation, some of the variability in ferritin levels reflects iron stores. These results highlight the complexity of interpreting interactions between ferritin and iron in the context of inflammation.

The respiratory failure and severe hypoxaemia observed in COVID-19 patients could contribute to the control of iron homeostasis through modulation of erythropoiesis and ERFE production. We found no association between ERFE and hepcidin or IL-6 suggesting that ERFE and erythropoiesis are not dominantly controlling hepcidin in this setting. However, we did find evidence of raised ERFE in patients with lower haemoglobin and serum iron, with a striking elevation in anaemic patients, suggesting that even in this setting of profound inflammation ERFE is raised in patients attempting to resolve a haemoglobin deficit, perhaps due to underlying iron deficiency.

The association of low serum iron with COVID-19 severity contrasts with bacterial sepsis where increased Tsat or serum iron associates with increased mortality, consistent with the hypoferremia of inflammation as an innate immune defence against siderophilic bacterial infection ( Arezes *et al*., 2015; Brandtner *et al*., 2020; Lan *et al*., 2018; Tacke *et al*., 2016). Interestingly in this study and previously we observe a positive association between blood lymphocyte counts and iron concentration ( Shah *et al*., 2020). Low serum iron availability impairs development of effective adaptive immunity to viral infection in animal models ( Frost *et al*., 2021; Preston *et al*., 2021). These observations warrant consideration of whether reduced iron availability could play a role in driving pathophysiology, and exploration of whether normalisation of iron status plays a role in the efficacy of therapeutics targeting innate immunity (such as Tocilizumab) and supports the funding of trials exploring iron supplementation in patients recovering from COVID-19 infection.

## Data availability

### Underlying data

The AspiFlu cohort is under licence to a third party (St George’s University of London). However, access to this can be arranged by contact with the AspiFlu team ( whurt@sgul.ac.uk, tbicanic@sgul.ac.uk). Interested readers or reviewers can contact the above to access the underlying data.

Zenodo: Underlying Data from DISCOVER cohort. https://doi.org/10.5281/zenodo.6587479 ( Hamilton, 2022).

This project contains the following underlying data:

- wellcome_open_res_data.rds

### Extended data

Zenodo: Underlying Data from DISCOVER cohort. https://doi.org/10.5281/zenodo.6587479 ( Hamilton, 2022).

This project contains the following extended data:

- S2_correlation_coefficents.csv

Data are available under the terms of the Creative Commons Attribution 4.0 International license (CC-BY 4.0).
