# Supplementary material for: Evaluation of perturbed iron-homeostasis in a prospective cohort of patients with COVID-19
Source: Wellcome Open Res. 2022 Jun 21;7:173. [Version 1] doi: 10.12688/wellcomeopenres.17904.1 (PMC9307999; doi:10.12688/wellcomeopenres.17904.1)
Supplement: Peer review history

# Peer review: Reviewer response for version 1

**DOI:** 10.21956/wellcomeopenres.19840.r51286

**Published:** 2022-07-22

**Authors:** Domenico Girelli

**Affiliations:** 1 Department of Medicine, Section of Internal Medicine, University of Verona, Verona, Italy

This is an interesting paper aimed to investigate iron metabolism in COVID-19.

 It is well written and the results substantially confirm some other previous reports, although it has to be taken into account that case series often differ in terms of pandemic waves, enrolment criteria, degree of severity of disease, vaccination status, and many other features.

 Below are my comments, aimed to help the authors to further improve the quality of the manuscript.   

1. Table 1 illustrates the characteristics of patients of the DISCOVER cohort. It is noteworthy that in a substantial fraction COVID-19 diagnosis was only clinically suspected but not proven through a molecular swab. This should be acknowledged in the discussion as a weakness (in the list of study’s limitations).
2. In the same line, on the "Results" section (page 5, first lines) it is stated that 15% of patients in the DISCOVER cohort resulted negative at the PCR test. As stated in the current version, it is unclear whether or not these negative patients were actually excluded from the analyses. If not, a clear explanation for such incongruence should be provided.
3. ERFE was measured in only one third of the patients from the DISCOVER cohort (n= 82, e.g. 246 minus 164). The same is true for hepcidin (lacking in 151; e.g. 61%). Although of great potential interest, such low numbers represent a weakness that needs to be clearly acknowledged.
4. Discussion, first sentence: it is stated that low serum iron levels are associated with a worse outcome and that this is in line with experimental work showing that iron deficiency is associated with less efficient immune response. Although fascinating, this is just an hypothesis that has been tested in a completely different experimental model. I would suggest not using this argument in such a strictly logical connection.   

Minor points:

- Table 1, first column: delete question comma after hypertension.
- suPAR is listed among inflammatory markers but it is not defined.

Is the work clearly and accurately presented and does it cite the current literature?

Yes

If applicable, is the statistical analysis and its interpretation appropriate?

Yes

Are all the source data underlying the results available to ensure full reproducibility?

No source data required

Is the study design appropriate and is the work technically sound?

Yes

Are the conclusions drawn adequately supported by the results?

Yes

Are sufficient details of methods and analysis provided to allow replication by others?

Yes

Reviewer Expertise:

iron metabolism.

I confirm that I have read this submission and believe that I have an appropriate level of expertise to confirm that it is of an acceptable scientific standard.

# Peer review: Reviewer response for version 1

**DOI:** 10.21956/wellcomeopenres.19840.r51283

**Published:** 2022-07-13

**Authors:** Günter Weiss

**Affiliations:** 1 Department of Internal Medicine II, Infectious Diseases, Immunology, Rheumatology, Medical University of Innsbruck, Innsbruck, Austria

This is an important and well-conducted retrospective study of a group with outstanding experience in iron homeostasis, immunology and infectious diseases, specifically with viral infections. The results are highly relevant for clinical estimation of the association between iron dys-homeostasis and course of SARS-CoV2 infection, also providing novel insigths into the dynamics of such alterations and their putative inter-relationship. I have only a few minor comments to this excellent piece of work.

- Minor: Table 1/Figure 1 - were these data obtained upon admission to the hospital? Please clarify in the legends.
- The authors describe that 15% had suspicion for COVID-19 but initially negative PCR tests. Were these patients tested PCR positive for SARS-CoV2 later on? If not, did the results, shown in table 1, differ, when such patients were excluded?
- The authors report on the unexpected results of low iron associated with high Erfe. However, it may well be that high Erfe is a consequence of increased Epo formation due to hypoxia/iron deficiency. In addition, the effects of Erfe on hepcidin regulation may be blunted due to stimulation of hepcidin expression by cytokines and/or cytokine mediated modulation of Erfe signaling in hepatocytes. The authors may comment on this. If Epo levels are available, their presentation would be of great interest.
- They authors may also emphasize their finding of a lacking association between alteration of iron/hematological parameters and persisting problems post COVID-19 (as shown by physical and mental composite scores).

Is the work clearly and accurately presented and does it cite the current literature?

Yes

If applicable, is the statistical analysis and its interpretation appropriate?

Yes

Are all the source data underlying the results available to ensure full reproducibility?

No source data required

Is the study design appropriate and is the work technically sound?

Yes

Are the conclusions drawn adequately supported by the results?

Yes

Are sufficient details of methods and analysis provided to allow replication by others?

Yes

Reviewer Expertise:

infectious dieases, immunology, iron metabolism, innate immunity

I confirm that I have read this submission and believe that I have an appropriate level of expertise to confirm that it is of an acceptable scientific standard.
